# Effects of Combined Epidural Anesthesia and General Anesthesia on Cognitive Function and Stress Responses of Elderly Patients Undergoing Liver Cancer Surgery

**DOI:** 10.1155/2021/8273722

**Published:** 2021-09-24

**Authors:** Zhixiu Meng, Cao Gao, Xin Li, Jiang Shen, Tao Hong, Xiaofeng He, Leijun Zhu

**Affiliations:** Department of Anesthesiology, The Third Affiliated Hospital of Soochow University, Changzhou 213003, Jiangsu, China

## Abstract

This study aimed at exploring the effects of combined epidural anesthesia and general anesthesia on the cognitive function and stress responses of elderly patients undergoing liver cancer surgery. One hundred and fifteen elderly patients were enrolled as research subjects. They were admitted to our hospital and underwent liver cancer surgery from August 2017 to May 2019. Fifty five cases were treated with general anesthesia (GA) (GA group), while the other sixty cases were treated with combined epidural anesthesia and general anesthesia (joint group). Scoring standards of Mini-Mental State Examination (MMSE) were used to evaluate the patients before and after operation. Their operating time, total fluid input (TFI), spontaneous breathing recovery time (SBRT), preoperative and postoperative indices of stress responses (epinephrine (EPI), cortisol (Cor), and norepinephrine (NE)), and postoperative adverse reactions were observed. There were statistically significant differences between the two groups with respect to anesthesia time, TFI, postoperative SBRT, and postoperative directional recovery time (DRT) (c*P* < 0.05). There was no difference in operating time, total fluid loss (TFL), and hospitalization time (*P* > 0.05). After operation, patients in both groups experienced a cognitive decline of different degrees and the MMSE scores decreased. There was no significant difference in the score between the two groups before operation and 3 days and 7 days after operation (*P* > 0.05). The score was significantly better in the joint group than that in the GA group at 6 hours and 1 day after operation (*P* < 0.05). There were no significant differences in levels of EPI, Cor, and NE between the two groups before operation (*P* > 0.05), but there were significant differences after operation. The total incidence of postoperative adverse reactions was 11.67% in the joint group and 25.45% in the GA group. In conclusion, combined epidural anesthesia and general anesthesia can significantly reduce postoperative cognitive dysfunction and inhibit postoperative stress responses in elderly patients undergoing liver cancer surgery. It has good application value in clinical practice.

## 1. Introduction

As a common malignant tumor in the digestive system, liver cancer has a high mortality rate and a natural survival period of less than 3–6 months [1]. According to statistics of a previous study, the disease is the fifth most common cancer worldwide [2]. It is estimated that there are approximately 780,000 new cases and 740,000 deaths globally every year. Cases in China alone account for 50% of the total number [3]. According to the National Cancer Center, the incidence of liver cancer ranked the second among all cancers in China in 2014 [4], and the incidence in the elderly has been gradually increasing because of the aging population in this country [5]. The global disease burden caused by the disease has resulted in losses of lives, and the disease is still an important public health problem in the world due to its high incidence, mortality rate, and aggressiveness.

The etiology and exact molecular mechanism of primary liver cancer are still unclear. According to epidemiological and experimental research data, hepatitis B virus (HBV) and hepatitis C virus (HCV) infection, aflatoxin, contaminated drinking water, and liver cirrhosis are all related to the pathogenesis of liver cancer [6]. The clinical symptoms of early liver cancer are not apparent, so most patients were already at the advanced stage once the symptoms appeared. After the early diagnosis of liver cancer, the disease is primarily treated by surgery with a high cure rate [7, 8]. However, the risk of surgical treatment rises with the increasing number of elderly patients. The patients' postoperative cognitive function and stress responses are different due to different anesthesia methods [9, 10]. Although general anesthesia (GA) is commonly used in liver cancer surgery, the incidence of postoperative cognitive dysfunction in elderly patients is high, which is also the focus of this study. Therefore, effects of combined epidural anesthesia and general anesthesia on the cognitive function and stress responses of elderly patients undergoing liver cancer surgery were explored in this study, so as to provide potential basis for future research.

## 2. Materials and Methods

### 2.1. Clinical Data

One hundred and fifteen elderly patients were enrolled as the research objects. They were admitted to The Third Affiliated Hospital of Soochow University, Changzhou, Jiangsu, China, and underwent liver cancer surgery from August 2017 to May 2019. Fifty five cases treated with GA were in the GA group, including 36 males and 19 females, with an average age of 67.5 ± 3.2 years. Sixty cases treated with combined epidural anesthesia and general anesthesia were in the joint group, including 33 males and 27 females, with an average age of 67.5 ± 3.1 years.

### 2.2. Inclusion and Exclusion Criteria

#### 2.2.1. Inclusion Criteria

Patients who met the diagnostic criteria for liver cancer in the guidelines from the National Comprehensive Cancer Network [11]; patients aged 60–77 years old; patients with complete medical records; patients with indications to GA and epidural anesthesia (EA); patients with educational level of primary school and above; patients willing to cooperate in investigation; patients without other serious organ diseases affecting this study. This study has been approved by the ethics committee of The Third Affiliated Hospital of Soochow University, Changzhou, Jiangsu, China, and all study participants provided written informed consent.

#### 2.2.2. Exclusion Criteria

Those with contraindications to anesthesia or liver cancer surgery; those who died during treatment; those with injury in important organs; those complicated with other tumors; those complicated with other cardiovascular and cerebrovascular diseases; those with physical disability; pregnant women; those complicated with other autoimmune diseases; those transferred to other hospitals; those with mental diseases, language dysfunction, or diseases affecting the results of this study.

### 2.3. Preoperative Preparation

Patients in the two groups fasted for 12 hours and were forbidden to drink water for 4 hours before operation. Venous infusion channels were established to carry out blood gas analysis and monitor vital signs and electrocardiograms of the patients.

### 2.4. Anesthesia Methods

Patients in the GA group were treated with GA. Anesthesia induction was first conducted with propofol (1.5 mg/kg) + sufentanil (0.2–0.6 *μ*g/kg) + atracurium besylate (0.2 mg/kg). Tracheal intubation was performed for mechanical ventilation, with tidal volume controlled at 8–10 mL/kg, respiratory frequency controlled at 10–12 times/min, and partial pressure end-tidal carbon dioxide maintained at 35–45 mmHg. During operation, the patients after sevoflurane inhalation were continuously administered with remifentanil and propofol by a micropump and intermittently injected intravenously with atracurium to keep the muscle relaxed and ensure the anesthetic effect. Drug dosage was determined by the patients' tolerance degree and stress responses, and their vital signs were closely monitored throughout the operation. Patients in the joint group were treated with combined epidural anesthesia and general anesthesia. Strict disinfection was carried out before puncture. The patients were placed in a lateral position (bend the knees and embrace the knees with both hands) to fully expose the puncture position. Thoracic 8–10 spinous process intervals were selected, which were commonly used puncture sites in the upper abdomen. Local anesthesia was conducted after the puncture position was determined. Lidocaine with a concentration of 1% was selected as the anesthetic drug. After the puncture position was confirmed in the epidural space, ropivacaine (20 mL) with a concentration of 5% was injected, and the needle was pulled out after the puncture. The anesthesia block level was maintained between *T*6 and *T*9. Mask oxygen inhalation was conducted, and GA was performed after the body position was maintained for 10–15 min. The anesthesia method was the same as that in the joint group. After operation, dicaine, flurbiprofen axetil, and granisetron were administrated in both groups for postoperative analgesia. The drug dosage was determined based on the patients' pain tolerance.

### 2.5. Scoring Standards

Mini-Mental State Examination (MMSE) was used as the scoring standards to evaluate the patients before operation and 6 h, 1 d, 3 d, and 7 d after operation. Its total score was 30 points, and a high score indicated a better cognitive function.

### 2.6. Outcome Measures

Main outcome measures: the patients' operating time, total fluid input (TFI), spontaneous breathing recovery time (SBRT), cognitive function at each time period, and preoperative and postoperative indices of stress responses were observed.

Secondary outcome measures: the patients' postoperative adverse reactions were observed.

### 2.7. Statistical Methods

In this study, SPSS20.0 (IBM Corp, Armonk, NY, USA) was used to statistically analyze the collected data. GraphPad 7 was used to plot the required figures. Kolmogorov–Smirnov (K-S) test was used to analyze the distribution of measurement data. The data conforming to normal distribution were expressed by mean ± standard deviation (Mean ± SD). Independent samples *t*-test was used for comparison between groups, while paired *t*-test was used for the comparison within groups. Count data were expressed by rate (%), analyzed by chi-square test, and represented by *χ*^2^. When *P* < 0.05, the difference was statistically significant.

## 3. Results

### 3.1. Clinical Data

Before operation, there were no significant differences between the GA and joint groups in terms of age, body mass index (BMI), MMSE score, serum alpha-fetoprotein (AFP), gender, place of residence, smoking, drinking, exercise habits, systolic blood pressure (SBP), and diastolic blood pressure (DBP), indicating comparability (*P* > 0.05). See Table 1.

### 3.2. Operating Time, TFI, and SBRT

There were statistically significant differences between the two groups with respect to anesthesia time, TFI, postoperative SBRT, and postoperative directional recovery time (DRT) (*P* < 0.05). There were no differences in operating time, total fluid loss (TFL), and hospitalization time (*P* > 0.05). See Table 2.

### 3.3. Cognitive Function at Different Time Periods

After the operation, patients in both groups experienced a cognitive decline of different degrees, and the MMSE scores decreased. There was no significant difference in the score between the two groups before operation and 3 days and 7 days after operation (*P* > 0.05), while the score was significantly better in the joint group than that in the GA group at 6 hours and 1 day after operation (*P* < 0.05). See Table 3.

### 3.4. Preoperative and Postoperative Indices of Stress Responses

**Figure 1:**
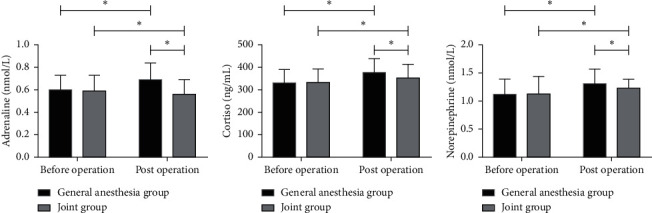
Indices of stress responses. (a) After operation, EPI level in the joint group decreased slightly and was lower than that in the GA group. (b) After operation, Cor level in the joint group increased slightly, but was lower than that in the GA group. (c) After operation, NE level in the joint group increased slightly, but was lower than that in the GA group. *Note*. ^*∗*^ indicates a difference between two groups (*P* < 0.05).

There were no significant differences in levels of epinephrine (EPI), cortisol (Cor), and norepinephrine (NE) between the two groups before operation (*P* > 0.05). There were significant differences after operation (*P* < 0.05). See Figure 1.

### 3.5. Postoperative Adverse Reactions

The total incidence of postoperative adverse reactions was 11.67% in the joint group and 25.45% in the GA group (*P* < 0.05). See Table 4.

## 4. Discussion

Liver cancer is mainly induced by alcohol, virus, and fatty liver injury [12]. Its early clinical manifestations are not apparent because of the decline of the sensory function and reaction ability in the elderly, so the disease is basically in the advanced stage when the patients feel the body abnormalities themselves, which definitely delays their treatment and increases the treatment difficulty [13]. Therefore, radiotherapy, chemotherapy, and surgery can be selected to relieve their pain and prolong their life [8, 14–16]. However, the operation is difficult for the elderly patients, because their surgical risk, postoperative cognitive dysfunction, and stress responses are higher than those of young patients with liver cancer. Currently, there are few studies available on the optimal anesthesia methods for the elderly patients. Therefore, in this study, effects of combined epidural anesthesia and general anesthesia on the cognitive function and stress responses of the elderly patients undergoing liver cancer surgery were explored, so as to provide a reference for clinical practice.

In this study, we first observed the intraoperative and postoperative recovery of the patients with liver cancer. There were statistically significant differences between the two groups with respect to anesthesia time, TFI, postoperative SBRT, and postoperative DRT. There were no differences in operating time, THL, and hospitalization time. This indicated that combined epidural anesthesia and general anesthesia could improve the patients' intraoperative and postoperative recovery capability. Liver cancer surgery requires a high level of anesthesia [17, 18], which is more obvious in elderly patients undergoing the surgery. The slight deviation of the level makes the patients prone to pulmonary stretch reflex [19], so it is difficult to achieve accurate anesthesia without affecting respiratory function. Scoring standards of MMSE were used to score the patients before and after operation. After operation, patients in both groups experienced cognitive decline of different degrees and decreasing MMSE scores. There was no significant difference in the score between the two groups before operation and 3 days and 7 days after operation. The score was significantly better in the joint group than that in the GA group at 6 hours and 1 day after operation. This suggested that combined epidural anesthesia and general anesthesia can improve the patients' postoperative cognitive function. We also observed the indices of stress responses before and after operation. There were no significant differences in levels of EPI, Cor, and NE between the two groups before operation, and there were significant differences after operation [20]. This demonstrated that combined epidural anesthesia and general anesthesia could stabilize the patients' indices of stress responses, while GA is slightly inferior. Finally, we observed the postoperative adverse reactions and found that their total incidence in the joint group was lower than that in the GA group. This further reveals the priority of combined epidural anesthesia and general anesthesia. Combined epidural anesthesia and general anesthesia can significantly reduce postoperative cognitive dysfunction and inhibit postoperative stress responses in elderly patients undergoing liver cancer surgery [21], so it has a good application value in clinical practice.

The abovementioned research has preliminarily suggested the effects of combined epidural anesthesia and general anesthesia on the cognitive function and stress responses of the elderly patients undergoing liver cancer surgery, which agree with the results of previous studies [22]. However, this study still has limitations. We did not take a series of neurocognitive tests. We did not follow up the patients for their prognosis. Therefore, we plan to perform more in-depth experimental analyses in future studies, so as to supplement our research results and provide services for clinical practice.

In summary, combined epidural anesthesia and general anesthesia is more effective than GA in reducing the postoperative cognitive dysfunction and stress responses of elderly patients undergoing liver cancer surgery, with low incidence of postoperative adverse reactions. Therefore, combined anesthesia, such as combined epidural anesthesia and general anesthesia can significantly reduce the postoperative cognitive dysfunction of the elderly patients undergoing liver cancer surgery.

## Figures and Tables

**Table 1:** Clinical information of patients (*n*(%)).

	GA group (*n* = 55)	Joint group (*n* = 60)	*χ*^2^ or *t*	*P* value
Age (years)	67.5 ± 3.2	67.5 ± 3.1	0.051	0.956
BMI (kg/cm^2^)	28.4 ± 2.61	27.85 ± 3.05	1.072	0.286
Gender			0.277	0.810
Male	36 (65.45)	33 (55.00)		
Female	19 (34.55)	27 (45.00)		
Place of residence			0.479	0.679
City	29 (52.72)	35 (58.83)		
Countryside	26 (47.28)	25 (41.67)		
Smoking			0.206	0.855
Yes	38 (69.09)	36 (60.00)		
No	17 (30.91)	24 (40.00)		
Drinking			0.117	0.917
Yes	39 (70.90)	48 (80.00)		
No	16 (29.10)	12 (20.00)		
Exercise habits			0.148	0.896
Yes	37 (67.27)	44 (73.33)		
No	18 (32.73)	16 (26.64)		
SBP (mmHg)	142.62 ± 12.87	138.86 ± 11.92	1.627	0.106
DBP (mmHg)	86.24 ± 8.67	85.16 ± 8.50	0.674	0.501
MMSE score	27.6 ± 2.83	27.74 ± 2.69	0.272	0.780
Serum AFP (ng/mL)	531 ± 69.20	523 ± 72.50	0.604	0.540

**Table 2:** Intraoperative and postoperative recovery.

	GA group (*n* = 55)	Joint group (*n* = 60)	*t*	*P* value
Operating time (min)	198.72 ± 56.14	187.39 ± 50.11	1.143	0.255
Anesthesia time (min)	214.67 ± 56.89	193.67 ± 55.73	2.073	0.040
TFI (mL)	1906.00 ± 201.00	1739.00 ± 182.00	3.192	0.002
TFL (mL)	375.73 ± 131.06	364.92 ± 120.73	0.460	0.646
Postoperative SBRT (min)	16.08 ± 3.62	13.87 ± 3.55	3.304	0.001
Postoperative DRT (min)	19.63 ± 4.01	17.36 ± 3.80	3.117	0.002
Hospitalization time (d)	18.55 ± 4.85	16.71 ± 5.31	1.934	0.056

**Table 3:** Cognitive function at different time periods.

	Before operation	6 hours after operation	1 day after operation	3 days after operation	7 days after operation
GA group (*n* = 55)	27.6 ± 2.83	21.77 ± 2.21	22.28 ± 2.51	26.45 ± 2.31	27.28 ± 2.21
Joint group (*n* = 60)	27.74 ± 2.69	23.58 ± 2.46	24.51 ± 2.58	27.09 ± 2.43	27.71 ± 2.11
*t*	0.272	4.173	4.691	1.000	1.067
*P* value	0.780	0.001	0.001	0.423	0.288

**Table 4:** Postoperative adverse reactions.

	GA group (*n* = 55)	Joint group (*n* = 60)	*χ*^2^/*t*	*P* value
Nausea	5(9.09)	1(1.67)	4.771	0.029
Vomiting	3(5.45)	1(1.67)		
Dyspnea	2(3.63)	2(3.33)		
Horner syndrome	4(7.27)	2(3.33)		
Total incidence	14(25.45)	6(11.67)		

## Data Availability

The datasets used and/or analyzed during the present study are available from the corresponding author on reasonable request.

## References

[1] Wong M. C. S., Jiang J. Y., Goggins W. B. (2017). International incidence and mortality trends of liver cancer: a global profile. *Scientific Reports*.

[2] Zhang X., Xu X., Ge G. (2019). MiR-498 inhibits the growth and metastasis of liver cancer by targeting ZEB2. *Oncology Reports*.

[3] Cong W.-M., Bu H., Chen J. (2016). Practice guidelines for the pathological diagnosis of primary liver cancer: 2015 update. *World Journal of Gastroenterology*.

[4] Chen W., Sun K., Zheng R. (2014). Cancer incidence and mortality in China. *Chinese Journal of Cancer Research*.

[5] Petrick J. L., Braunlin M., Laversanne M., Valery P. C., Bray F., McGlynn K. A. (2016). International trends in liver cancer incidence, overall and by histologic subtype, 1978-2007. *International Journal of Cancer*.

[6] Minemura M., Shimizu Y. (2015). Gut microbiota and liver diseases. *World Journal of Gastroenterology*.

[7] Chatterjee R., Mitra A. (2015). An overview of effective therapies and recent advances in biomarkers for chronic liver diseases and associated liver cancer. *International Immunopharmacology*.

[8] Vitale A., Burra P., Frigo A. C. (2015). Survival benefit of liver resection for patients with hepatocellular carcinoma across different Barcelona Clinic Liver Cancer stages: a multicentre study. *Journal of Hepatology*.

[9] Cassinello F., Prieto I., del Olmo M., Rivas S., Strichartz G. R. (2015). Cancer surgery: how may anesthesia influence outcome?. *Journal of Clinical Anesthesia*.

[10] Ni C. Y., Yang Y., Chang Y. Q. (2013). Fast-track surgery improves postoperative recovery in patients undergoing partial hepatectomy for primary liver cancer: a prospective randomized controlled trial. *European Journal of Surgical Oncology (EJSO)*.

[11] Wald C., Russo M. W., Heimbach J. K., Hussain H. K., Pomfret E. A., Bruix J. (2013). New OPTN/UNOS policy for liver transplant allocation: standardization of liver imaging, diagnosis, classification, and reporting of hepatocellular carcinoma. *Radiology*.

[12] Marengo A., Rosso C., Bugianesi E. (2016). Liver cancer: connections with obesity, fatty liver, and cirrhosis. *Annual Review of Medicine*.

[13] Zhang W., Sun B. (2015). Impact of age on the survival of patients with liver cancer: an analysis of 27,255 patients in the SEER database. *Oncotarget*.

[14] Sun J.-H., Luo Q., Liu L.-L., Song G.-B. (2016). Liver cancer stem cell markers: progression and therapeutic implications. *World Journal of Gastroenterology*.

[15] Shao D., Li J., Zheng X. (2016). Janus “nano-bullets” for magnetic targeting liver cancer chemotherapy. *Biomaterials*.

[16] Su T.-S., Liang P., Liang J. (2017). Long-term survival analysis of stereotactic ablative radiotherapy versus liver resection for small hepatocellular carcinoma. *International Journal of Radiation Oncology, Biology, Physics*.

[17] Pathak S., Hakeem A., Pike T. (2015). Anaesthetic and pharmacological techniques to decrease blood loss in liver surgery: a systematic review. *ANZ Journal of Surgery*.

[18] De La Serna S., Vilana R., Sánchez-Cabús S. (2015). Results of laparoscopic radiofrequency ablation for HCC. Could the location of the tumour influence a complete response to treatment? A single European centre experience. *International Hepato-Pancreato-Biliary Association*.

[19] Terao M., Takaki A., Maruyama T. (2015). Serum oxidative/anti-oxidative stress balance is dysregulated in potentially pulmonary hypertensive patients with liver cirrhosis: a case control study. *Internal Medicine*.

[20] Su Y., Pu Y., Zhao Z., Yang X. (2020). Influence of combined epidural anesthesia on cognitive function, inflammation and stress response in elderly liver cancer patients undergoing surgery. *Oncology Letters*.

[21] Hadimioglu N., Ulugol H., Akbas H., Coskunfirat N., Ertug Z., Dinckan A. (2012). Combination of epidural anesthesia and general anesthesia attenuates stress response to renal transplantation surgery. *Transplantation Proceedings*.

[22] Zhu J., Zhang X. R., Yang H. (2017). Effects of combined epidural and general anesthesia on intraoperative hemodynamic responses, postoperative cellular immunity, and prognosis in patients with gallbladder cancer. *Medicine*.

